# Supplementary material for: Using Deauville Scoring to Guide Consolidative Radiotherapy in Diffuse Large B-Cell Lymphoma
Source: Cancers (Basel). 2024 Sep 27;16(19):3311. doi: 10.3390/cancers16193311 (PMC11475697; doi:10.3390/cancers16193311)
Supplement: Supplementary file 1 [file cancers-16-03311-s001.zip › cancers-3198501-supplementary.pdf]

Supplementary Materials

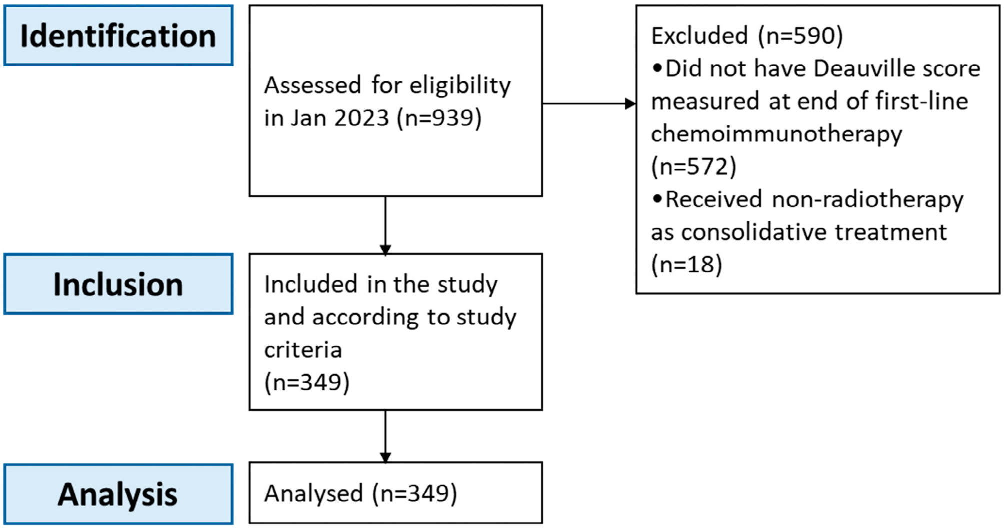

Supplementary Figure S1. STROBE flowchart of included patients.

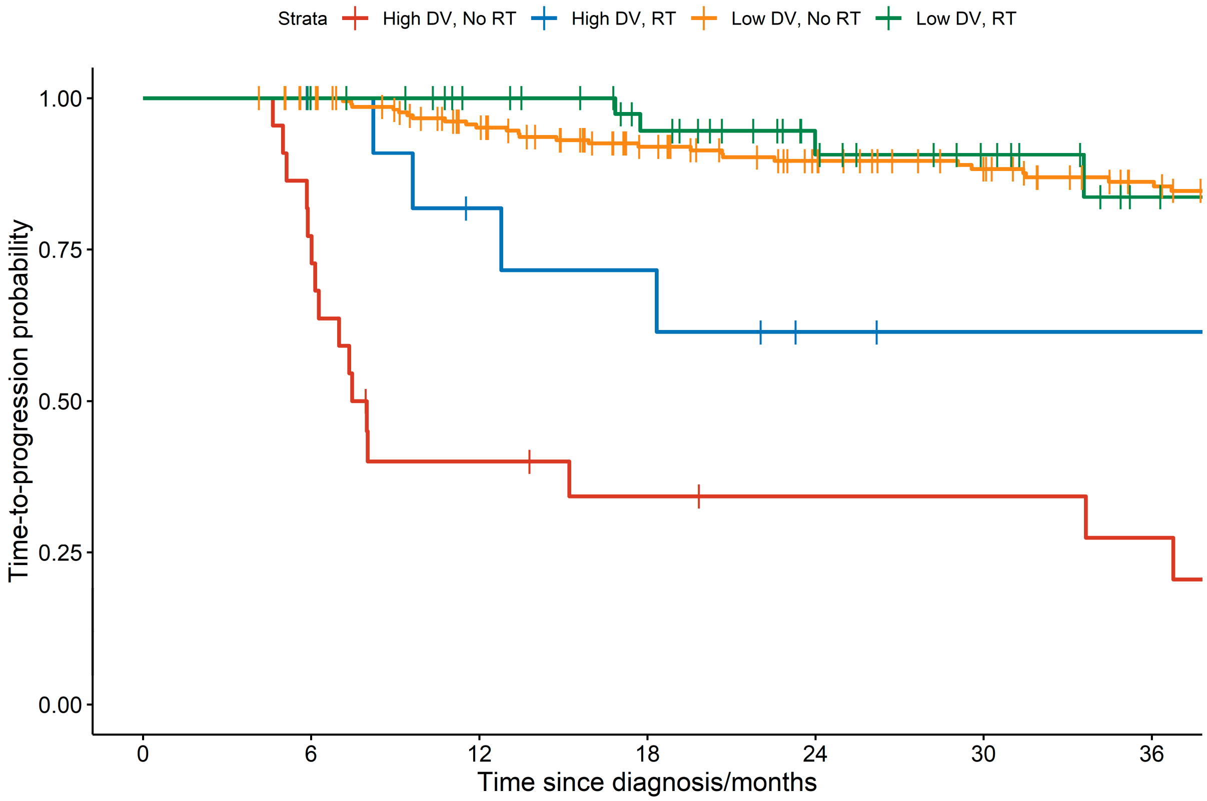

Number at risk

High DV, No RT	22	17	8	6	5	5	4
High DV, RT	12	11	8	7	4	3	3
Low DV, No RT	221	215	189	164	144	132	116
Low DV, RT	51	49	43	34	23	17	9

Supplementary Figure S2. Combined graph by Deauville and radiotherapy status of patients who received ≥6 cycles of chemoimmunotherapy, and did not progress before the end of the chemoimmunotherapy regimen, with TTP measured from date of diagnosis.

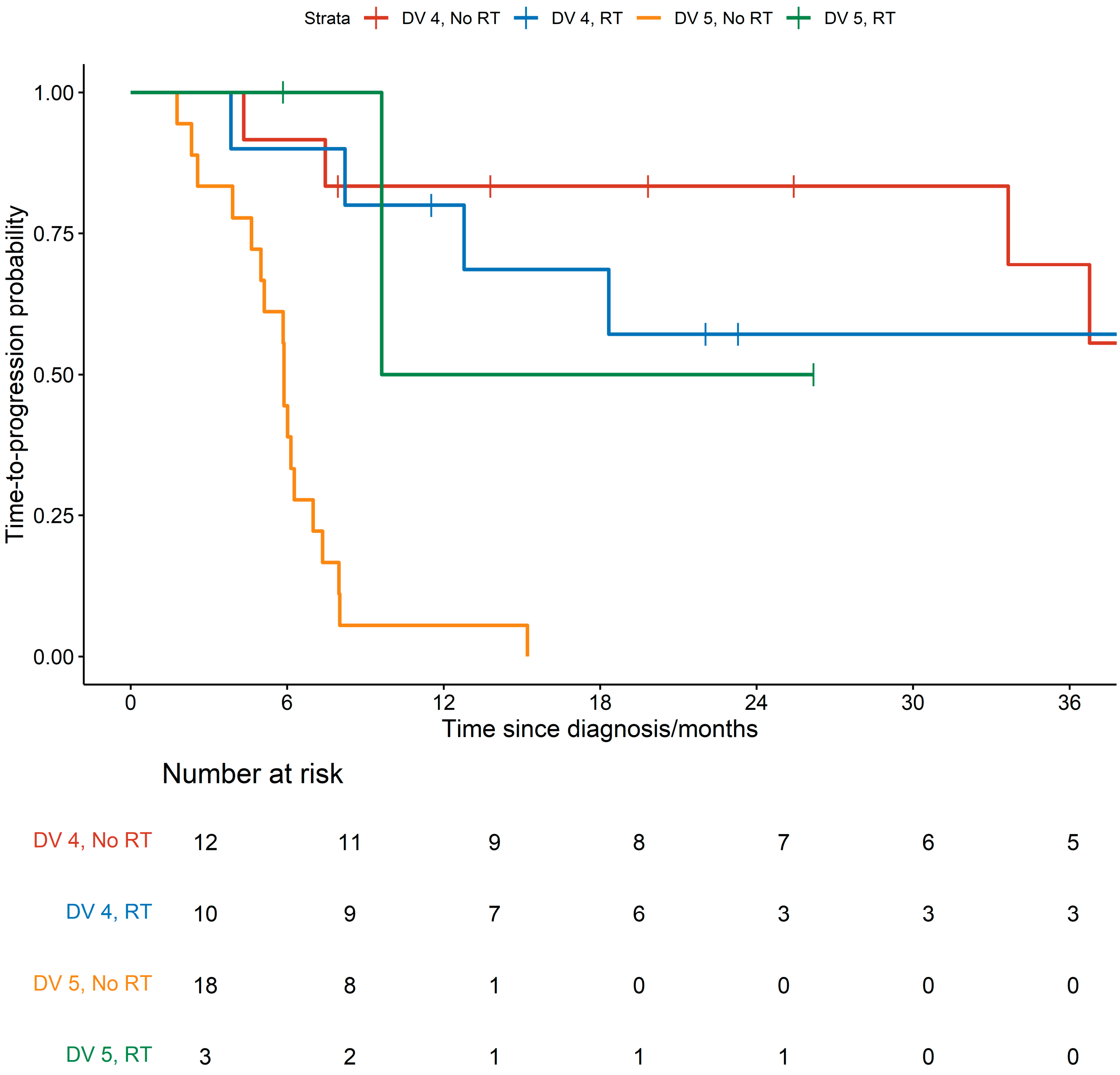

Supplementary Figure S3. Combined graph by Deauville and radiotherapy status of DV4 and DV5 patients.

Supplementary Table S1. Multivariable analysis of baseline characteristics associated with time-to-progression events in patients who received ≥6 cycles of chemoimmunotherapy, and did not progress before the end of the chemoimmunotherapy regimen.

**Characteristic**	**HR**	**95% CI**	**p-value**
Receipt of Consolidative RT			0.018
RT-omitted	1.00 (ref)	—	
RT-treated	0.274	0.085, 0.876	
Deauville Score			0.000
DV4-5	1.00 (ref)	—	
DV1-3	0.115	0.058, 0.232	
Marrow Involvement			0.252
No	1.00 (ref)	—	
Yes	1.656	0.791, 3.469	
Unavailable	3.889	0.518, 29.21	
Haemoglobin Levels	0.983	0.852, 1.135	0.820
Presence of B Symptoms			0.076
No	1.00 (ref)	—	
Yes	1.728	0.945, 3.161	
Unavailable	0.754	0.348, 1.632	
International Prognostic Index			0.241
High >=3	1.00 (ref)	—	
Low <3	0.725	0.404, 1.300	
Unavailable	0.000	0.000, Inf	
Interaction term between Receipt of RT and Deauville Score	2.209	0.468, 10.44	0.321

Supplementary Table S2. Multivariable interaction analysis in patients who received ≥6 cycles of chemoimmunotherapy, and did not progress before the end of the chemoimmunotherapy regimen.

	Multivariable-adjusted model^		
	**HR (95% CI)**	**P**	**P(Int*)**
Overall: RT-treated vs RT-omitted	0.71 (0.33, 1.51)	0.350	-
DV4-5: RT-treated vs RT-omitted	0.27 (0.09, 0.88)	0.029	0.321
DV1-3: RT-treated vs RT-omitted	0.60 (0.21, 1.72)	0.345	

^ Adjusted for International Prognostic Index, presence of B symptoms, bone marrow involvement, and hemoglobin levels. * Interaction term between RT and DV risk groups.
